# Anesthetic Considerations for Cesarean Delivery in a Patient With Third-Degree Heart Block: A Case Report

**DOI:** 10.7759/cureus.80207

**Published:** 2025-03-07

**Authors:** Kirti Rishi, Mohamed A. Ibrahim

**Affiliations:** 1 Anesthesiology, University of Texas Medical Branch, Galveston, USA

**Keywords:** arrythmia, cardiac arrythmia, cesarean section (cs), complete heart block in pregnancy, obstetric anesthesia, third-degree heart block

## Abstract

Congenital third-degree complete heart block (CHB) detected during pregnancy is a rare condition. This report discusses a pregnant patient with an incidental finding of CHB and its implications for maternal and fetal outcomes. A 21-year-old female patient, gravida 2 para 0 (G2P0010), first registered at five weeks, two days gestation, with an incidental finding of third-degree heart block. Her baseline heart rate of 40-50 beats per minute, with no prior cardiac diagnosis. She had a history of miscarriage at six weeks' gestation. During the current pregnancy, she experienced two episodes of dizziness upon standing, each resolving within a minute without signs of hemodynamic instability. A 12-lead EKG and 24-hour Holter monitoring confirmed CHB, and echocardiography ruled out secondary causes. Cardiology and electrophysiology recommended temporary transcutaneous pacing and bedside atropine in case of instability. CHB in pregnancy is often congenital and characterized by independent ventricular activity due to atrial stimulus blockage. While typically asymptomatic, symptoms such as dizziness, hypotension, syncope, severe bradycardia, and cardiac arrest can occur. Pregnancy and labor stress, including the Valsalva maneuver, can exacerbate bradyarrhythmia, leading to adverse outcomes. Inadequate fetal perfusion and oxygenation can result in fetal bradycardia and hypoxia. Management requires a multidisciplinary approach, with echocardiograms, Holter monitoring, and, in some cases, cardiac MRI to rule out structural heart disease. Asymptomatic patients with good functional capacity may avoid permanent pacemakers, though temporary pacing is considered on a case-by-case basis. Neuraxial anesthesia is preferred for cesarean delivery in both symptomatic and asymptomatic CHB patients due to its minimal impact on myocardial function. General anesthesia should be avoided when possible. If necessary, anesthetic agents with minimal cardiac depression, such as ketamine, etomidate, rocuronium, and isoflurane, are recommended. Assisted early deliveries, such as vacuum or forceps, can help reduce the risk of Valsalva-induced bradycardia. Asymptomatic CHB cases without significant heart disease typically have favorable outcomes. However, careful cardiovascular monitoring and individualized care plans are essential to mitigate potential complications. Postpartum cardiology follow-up is necessary to assess the development of new symptoms and determine the need for a permanent pacemaker. This case highlights the importance of early diagnosis, adequate monitoring, early elective delivery, and multidisciplinary management in CHB during pregnancy. Neuraxial anesthesia and strategic labor management are key to ensuring positive maternal and fetal outcomes. Further research is needed to develop standardized guidelines for this rare condition.

## Introduction

A heart block is a dysfunction in the heart's electrical conduction system, which is responsible for regulating the rhythmic contraction of the ventricles. This condition leads to ineffective ventricular contractions and insufficient blood flow to the systemic circulation. In a complete heart block (CHB), there is an absence of supraventricular impulse transmission to the ventricles, resulting in the dissociation of atrioventricular activity. This causes independent atrial and ventricular contractions, leading to bradycardia. The cardiac rhythm is maintained by a junctional or a ventricular escape rhythm. CHB can be congenital or acquired. Acquired causes include myocardial infarction, cardiac procedures, valvular disorders, viral myocarditis, and transplacental transfer of antibodies (e.g., anti-Ro/SSA and anti-La/SSB) in autoimmune diseases like systemic lupus erythematosus (SLE) and Sjogren's syndrome. It can also result from endocrine disorders (pituitary, adrenal, thyroid, and parathyroid glands), toxins (e.g., lead, copper, and arsenic), drug toxicity (e.g., beta-blockers, calcium channel blockers, and digoxin), or electrolyte abnormalities such as hyperkalemia, hypocalcemia, and hypermagnesemia. CHB is rare, occurring in approximately 1 in 22,000 live births [1]. Managing patients with CHB who require a caesarian section presents unique challenges for anesthesiologists due to the risk of severe bradycardia, hypotension, syncope due to ventricular standstill, sudden cardiac arrest, and hemodynamic instability. Careful selection of anesthesia, anesthetic agents, invasive arterial blood pressure (BP) monitoring, cardiac monitoring, and backup pacing are essential for optimal hemodynamics. A multidisciplinary team, including obstetricians, cardiologists, electrophysiologists, and anesthesiologists, is vital to ensure the safety of both the mother and the baby.

## Case presentation

**Figure 1:**
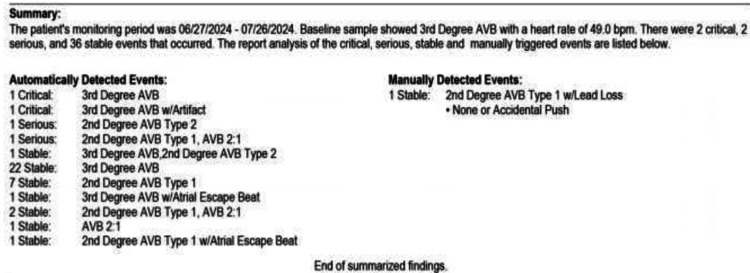
Holter monitoring summary indicating heart block in the patient.

**Figure 2:**
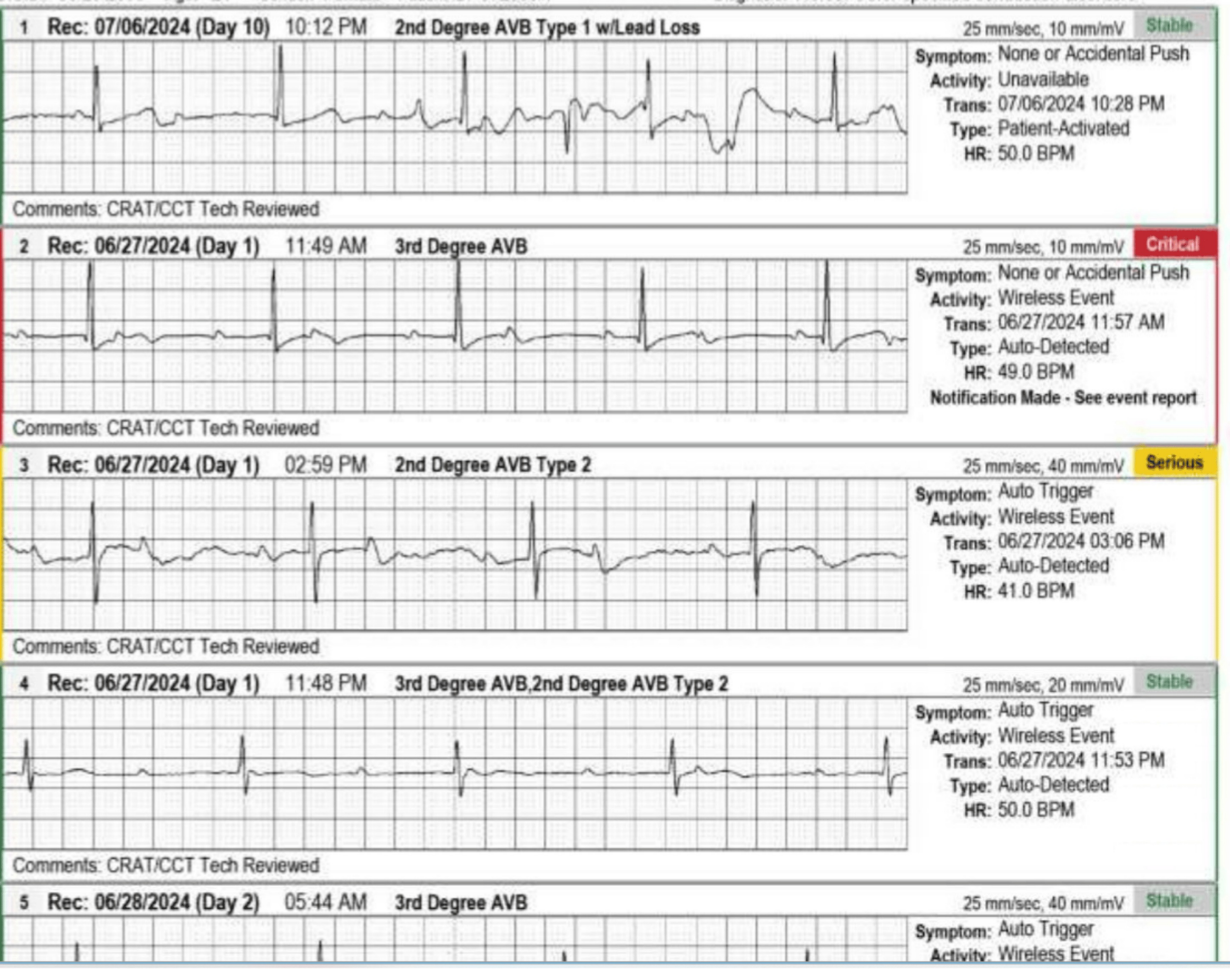
Holter findings indicating heart block in the patient.

**Figure 3:**
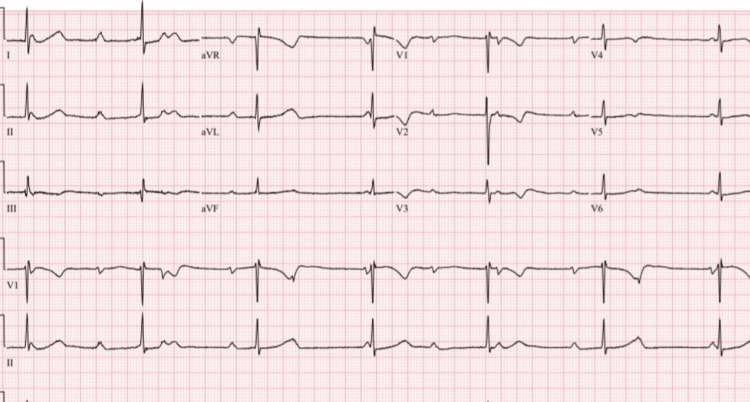
ECG of the patient showing sinus rhythm with complete dissociation of P-waves from QRS complexes, indicating complete heart block with junctional bradyarrhythmia. Ventricular rate: 40 beats per minute. QRS duration: 74 ms. QT/QTcB: 560/456 ms. P-R-T axes: 19°-38°-19°.

This case describes a 21-year-old female patient, G2P0010, with an incidental finding of a congenital third-degree CHB. Her baseline heart rate was 40-50 beats per minute, with a metabolic equivalent (MET) of 5, a baseline BP (taken in a sitting position with a cuff on the left arm) of 109/59 mmHg, a height of 60 inches, and a BMI of 25 kg/m^2^. Cardiovascular examination revealed normal S1 and S2 heart sounds, with no murmurs, rubs, gallops, or pedal edema. The patient had a history of one miscarriage at six weeks' gestation but no prior complaints of chest pain, shortness of breath, palpitations, or syncope. Her medical history was nonsignificant, with no history of drug abuse or use of antiarrhythmic medications (e.g., beta-blockers, calcium-channel blockers, or digitalis). Secondary causes of heart block, such as Lyme disease, SLE, toxins, cardiomyopathy, valvular diseases, myocardial ischemia, or prior heart surgeries, were ruled out. There was no notable family history. She had experienced two episodes of dizziness during pregnancy, triggered by standing up from a sitting position. Cardiology and electrophysiology were consulted. The patient was placed on a Holter monitor for 30 days, which revealed heart rate fluctuations from 37 to 50 beats per minute (Figure 1 and Figure 2), with corresponding BP changes. An EKG confirmed a complete atrioventricular (AV) heart block with complete dissociation of P-waves from QRS complexes and junctional bradyarrhythmia (Figure 3).

The transthoracic echocardiogram revealed normal cardiac structure and function, with an ejection fraction of 55%-60%, normal systolic and diastolic functions, and normal left (LV) and right ventricular (RV) sizes. There were no septal or regional wall motion abnormalities. The aortic and mitral valves appeared normal, with trace mitral and tricuspid regurgitations. Regarding her delivery plan, cardiology recommended placing temporary transcutaneous pacer pads and having bedside atropine available to prevent bradycardia in case the patient becomes hemodynamically unstable or develops dizziness, syncope, or altered mentation. 

Labor analgesia was provided using an epidural infusion pump, delivering 0.2% ropivacaine at 10 mL/hour via a catheter. At the patient’s request, an elective cesarean section was performed in the morning, utilizing the epidural catheter. The risks and benefits of prophylactic temporary pacing were discussed with the patient. Preoperatively, two large-bore 16-gauge IV cannulas were inserted for adequate IV access, and a 20G arterial line was placed in the right radial artery for invasive heart rate and BP monitoring. In the operating room, 7.5 mL of 0.5% bupivacaine and 7.5 mL of 2% lidocaine were administered through the epidural catheter in 5 mL increments to achieve sufficient motor and sensory blocks up to the T4 level, while maintaining optimal BP. To prevent bradycardia, 0.2 mg glycopyrrolate was administered IV as a prophylactic measure, and an epinephrine infusion of 16 mcg/mL IV was kept on hand for emergency use. Standard ASA monitors and pacer pads were attached to the defibrillator machine. Two episodes of hypotension occurred, which were unrelated to the epidural medication, and were treated with 6 mg of IV ephedrine each time. The patient's vitals remained stable throughout the surgery, with a heart rate of 40-50 beats per minute (average 47 beats per minute) and an average BP of 109/78 mmHg.

The baby was delivered without complications. After delivery, our team administered 3 mg of preservative-free morphine with 5 mL of 2% lidocaine through the epidural catheter. The patient had an uncomplicated postoperative course. The baby’s Apgar scores were 8 and 9 at one and five minutes, respectively. Neonatal monitoring was performed due to the maternal intraoperative administration of opioids. A neonatal EKG was conducted after birth and did not detect any cardiac arrhythmias. Postpartum, the patient was followed by cardiology and electrophysiology to assess for any new cardiac symptoms related to the heart block. Two months after delivery, the patient reported gradual shortness of breath on exertion. After discussing the risks and benefits, a permanent pacemaker was placed by cardiology.

## Discussion

CHB is a disorder of the cardiac conduction system characterized by a complete absence of conduction between the atria and ventricles [1]. Congenital CHB has an unknown etiology but is associated with maternal connective tissue diseases, in utero exposure to lupus antibodies, and complex congenital heart disease [2]. Acquired causes in young adults include prior cardiac surgery (e.g., ventricular/atrial septal defect repair), valvular diseases, cardiomyopathy, myocardial ischemia, myocardial fibrosis, transplacental transfer of autoimmune diseases (e.g., SLE and Sjogren's disease), toxins, drug-induced causes, electrolyte abnormalities, and complex congenital heart disease. CHB detected for the first time during pregnancy and delivery occurs in 1 in 20,000 live births [3]. The most common cause of CHB is congenital. It presents with a complete absence of stimulus transmission from the atria to the ventricles, leading to independent atrial and ventricular activities on the EKG, with no relation between the P-wave and the QRS complex. Depending on the location of the heart block (above or below the bundle of His), the patient may be symptomatic and atropine-nonresponsive or asymptomatic and responsive to atropine and sympathomimetic drugs. The EKG may show a normal-width, narrow QRS complex (junctional escape rhythm) or a wide QRS complex (ventricular escape rhythm). Junctional or ventricular escape rhythms arise when the rate of supraventricular impulses from the AV node or ventricle is less than the intrinsic rate of the ectopic pacemaker. In CHB with a wide QRS complex (>120 ms, ventricular escape rhythm), the rhythm is less stable than in CHB with a narrow QRS, as the escape pacemaker is less consistent and slower, typically pacing at 20-40 beats per minute. This slow rate increases the risk of asystole and sinus arrest/pause and may be associated with left bundle branch block (LBBB) or right bundle branch block (RBBB) morphology, resulting in poor perfusion and potential fetal hypoxia.

Patients with CHB can be asymptomatic; however, symptoms may develop later in life due to the variable degree of heart block [4]. The feto-maternal outcome is generally favorable in cases of uncomplicated bradyarrhythmia and asymptomatic patients. A slow maternal heart rate (<50 beats per minute) may lead to fetal complications such as hydrops fetalis, preterm birth, intrauterine growth restriction, neonatal heart failure, or exercise intolerance in the child. Asymptomatic pregnant patients without pacemakers may experience sudden cardiac arrest, heart failure, or an inability to maintain cardiac output for sufficient fetal perfusion during pregnancy. Additionally, they may become symptomatic during labor due to Valsalva-induced bradycardia [5,6]. Tachycardia cannot occur in these patients during labor due to CHB, preventing the attainment of the cardiac output necessary to maintain hemodynamics for safe delivery. Cardiac pacemakers should be implanted in symptomatic patients at the time of diagnosis and during pregnancy [7]. In contrast, prophylactic pacemakers are not required in asymptomatic patients; however, temporary pacemakers may be inserted during labor and delivery to maintain heart rate and hemodynamic stability [8]. In emergency situations, transcutaneous pacing should be initiated until transvenous pacing is possible.

There are several considerations for patients with complete AV heart block who require surgery. First, a thorough preoperative evaluation should focus on the cardiovascular history. A 12-lead EKG and echocardiogram are mandatory to rule out structural heart disease and evaluate the ejection fraction, especially if a cardiovascular implantable electronic device (CIED) is being considered [9]. A cardiac MRI may also be necessary to rule out myocardial fibrosis and infiltrative disorders. Patients with heart block undergoing surgeries are at risk of resistant bradycardia, hypotension, arrhythmias, cardiac arrest, Stokes-Adams attacks, and sudden death [10]. Patients reporting dizziness, shortness of breath, and exertional syncope, especially during pregnancy, may have a higher risk of decompensation and may require a pacemaker. In addition, any procedure to reduce the second stage of labor, such as forceps or vacuum assistance, is preferred to avoid the Valsalva maneuver, which can induce reflex bradycardia [11].   

In asymptomatic patients with good functional capacity, permanent pacemaker placement may be avoided, and the need for temporary pacing should be determined on a case-by-case basis. Our patient had good functional capacity and remained asymptomatic even in early labor, so we decided to have temporary pacing equipment available, with cardiology services on standby. Transcutaneous pacing pads were applied if needed until transvenous pacing could be initiated. 

Neuraxial anesthesia is the preferred choice for caesarian sections in pregnant patients with heart block. Many drugs used in general anesthesia can directly depress myocardial contractility, reduce cardiac output, and exacerbate preexisting conduction defects. Propofol has dose-dependent negative inotropic and vasodilatory effects. When infused rapidly or at doses greater than 8 mg/kg, it can diminish voltage-dependent L-type calcium channels [12], which play an important role in the pacemaker activity in nodal cells, potentially leading to torsades de pointes. Ketamine has a dose-dependent effect on the heart, and in higher doses, it can increase the cardiac oxygen demand, making it undesirable in these patients. Volatile anesthetics such as sevoflurane and isoflurane can enhance AV nodal block in the presence of preexisting conduction abnormalities [13]. They can prolong cardiac conduction and significantly extend the QT interval by inhibiting voltage-gated sodium and L-type calcium channels, potentially leading to fatal torsades de pointes. Amide local anesthetics like lidocaine, bupivacaine, and ropivacaine are sodium channel blockers and should be used with caution. Succinylcholine can increase the QTc interval, especially when combined with thiopentone, potentially exacerbating AV blocks [14].  AV nodal-blocking drugs, such as adenosine, beta-blockers, calcium channel blockers, flecainide, amiodarone, and digoxin, should be avoided [15]. High-dose opioids may cause bradycardia and hypotension and should be used with care. QT-prolonging agents, like ondansetron and metoclopramide, can worsen heart block and should be avoided [16]. Vecuronium has been associated with asystole in patients with conduction defects [17].

In our patient, the diagnosis of CHB was identified at near-term pregnancy, with a heart rate of 40-50 beats per minute. An elective cesarean section was performed under epidural anesthesia, resulting in slow hemodynamic changes that allowed our team to manage the situation promptly. 

Neuraxial anesthesia is the preferred anesthetic approach for cesarean delivery, including in patients with Modified World Health Organization Classification of Cardiovascular Disease in Pregnancy class III or IV lesions [18]. Stable hemodynamics can be maintained through continuous invasive monitoring, adequate intravascular support, and the use of IV ephedrine as needed. Indications for general anesthesia include cardiopulmonary decompensation requiring intubation or contraindications to neuraxial anesthesia, such as anticoagulation, thrombocytopenia, or maternal refusal. When general anesthesia is required, drugs with cardiodepressant effects should be avoided. Preferred drugs for induction include ketamine, while rocuronium is used for muscle relaxation. Careful use of volatile anesthetics for maintenance, with aggressive hemodynamic monitoring, is essential. Post-delivery maternal evaluations, such as EKG, echocardiography, and assessment for a permanent pacemaker, are critical for the management of CHB patients. 

## Conclusions

Neuraxial anesthesia, in conjunction with temporary transcutaneous pacing and emergency cardiac medications, provides a safe and effective option for cesarean deliveries in pregnant patients with CHB, whether symptomatic or asymptomatic. To optimize outcomes, a multidisciplinary approach is imperative. Prompt recognition and thorough evaluation of this condition during pregnancy are crucial. Furthermore, close monitoring, elective pacemaker placement when necessary, and consideration of earlier delivery can significantly enhance safety. Appropriate invasive arterial BP and cardiac monitoring are critical, as is a diligent postpartum follow-up to assess cardiac function and determine the need for a permanent pacemaker. By implementing these essential interventions throughout the preoperative, labor, and perioperative periods, we can greatly safeguard the health and well-being of parturients with CHB. 
